# Supplementary material for: Identification of Prognosis-Related Oxidative Stress Model with Immunosuppression in HCC
Source: Biomedicines. 2023 Feb 24;11(3):695. doi: 10.3390/biomedicines11030695 (PMC10045103; doi:10.3390/biomedicines11030695)
Supplement: Supplementary file 1 [file biomedicines-11-00695-s001.zip › biomedicines-2119372-supplementary.pdf]

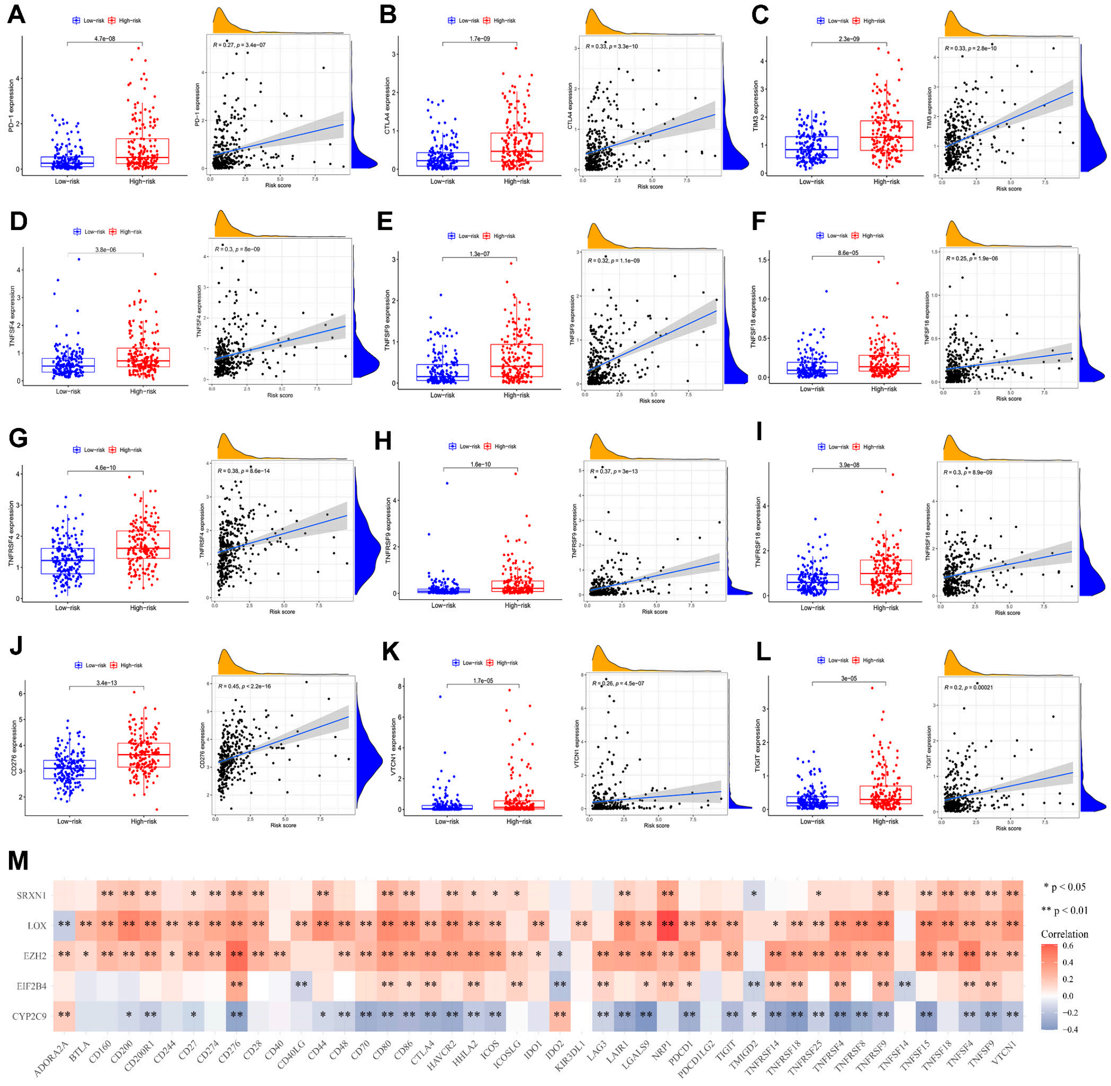

Figure S1. Associations between risk model and immune checkpoint genes. **(A-L)** Expression of PD-1, CTLA4, TIM3, CD276, TNFSF4,TNFSF9, TNFSF18, TNFRSF4, TNFRSF9, TNFSF18,CD276,VTCN1, and TIGIT in different groups of HCC patients and the correlation of riskscore and genes mentioned above. **(M)** Relationship of model genes and several checkpoint genes. $*p < 0.05$; $**p < 0.01$

Table S1. DEOSGs between normal liver and HCC samples in TCGA-LIHC dataset

gene	conMean	treatMean	logFC	pValue	fdr
HAMP	148.2531	15.96737	-3.21486	6.51×10^{-25}	1.26×10^{-23}
CYP2C19	3.085339	0.382142	-3.01325	3.19×10^{-17}	1.54×10^{-16}
VIPR1	3.069224	0.402004	-2.93259	3.31×10^{-28}	4.17×10^{-26}
PVALB	1.048306	0.140464	-2.89979	8.21×10^{-27}	3.49×10^{-25}
NTF3	1.419535	0.194418	-2.86819	1.89×10^{-27}	1.08×10^{-25}
CYP1A2	162.061	24.79812	-2.70823	4.83×10^{-24}	7.59×10^{-23}
DBH	10.7885	1.80472	-2.57965	1.78×10^{-24}	3.04×10^{-23}
FOS	113.2005	21.1632	-2.41925	6.68×10^{-21}	5.24×10^{-20}
NAT2	25.52141	4.83393	-2.40044	1.20×10^{-25}	3.13×10^{-24}
CSF3	0.0673	0.012922	-2.38077	2.67×10^{-7}	4.72×10^{-7}
ADAMTS13	5.109747	1.120561	-2.18903	5.33×10^{-28}	4.48×10^{-26}
CYP2C8	449.4618	100.5748	-2.15993	6.04×10^{-26}	1.69×10^{-24}

CYP2B6	126.864	29.24442	-2.11705	1.14×10^{-20}	8.54×10^{-20}
CXCL12	30.48354	7.355334	-2.05117	1.08×10^{-24}	1.97×10^{-23}
LCAT	68.11541	16.82336	-2.01752	2.17×10^{-26}	7.54×10^{-25}
EGR1	126.0517	32.6503	-1.94885	4.40×10^{-19}	2.67×10^{-18}
ESR1	2.143048	0.557843	-1.94173	1.04×10^{-22}	1.14×10^{-21}
LPA	12.53768	3.592235	-1.80332	2.38×10^{-22}	2.48×10^{-21}
RCAN1	22.9489	6.602392	-1.79736	5.46×10^{-24}	8.34×10^{-23}
PTGS2	0.685695	0.20982	-1.70841	5.31×10^{-17}	2.41×10^{-16}
GRIN2B	0.075976	0.023391	-1.69959	1.04×10^{-16}	4.57×10^{-16}
SOCS3	40.47743	12.83836	-1.65666	4.00×10^{-10}	9.00×10^{-10}
HBB	40.93615	13.23047	-1.62951	1.47×10^{-16}	6.41×10^{-16}
HP	3721.999	1270.449	-1.55074	6.60×10^{-21}	5.22×10^{-20}
SERPINE1	129.2231	45.04283	-1.52049	2.29×10^{-11}	5.75×10^{-11}
IL1RN	42.18082	14.71118	-1.51967	2.19×10^{-11}	5.54×10^{-11}
AMPD1	0.080093	0.027935	-1.51963	1.41×10^{-12}	3.99×10^{-12}
CXCR1	0.379962	0.13576	-1.4848	1.19×10^{-14}	4.04×10^{-14}
IL6	0.867032	0.320722	-1.43476	3.10×10^{-5}	4.64×10^{-5}
GLS2	4.778707	1.786804	-1.41924	1.24×10^{-15}	4.83×10^{-15}
HGF	2.775148	1.062623	-1.38493	3.52×10^{-20}	2.45×10^{-19}
GADD45B	138.0002	53.07551	-1.37855	6.51×10^{-15}	2.32×10^{-14}
NGFR	6.559445	2.597936	-1.33621	8.26×10^{-18}	4.34×10^{-17}
CR1	0.217472	0.086332	-1.33286	3.83×10^{-12}	1.05×10^{-11}
IL1RAPL2	0.267202	0.106925	-1.32133	1.08×10^{-13}	3.38×10^{-13}
TH	0.103477	0.041963	-1.30212	7.05×10^{-23}	8.24×10^{-22}
CYP2C9	287.5833	118.3821	-1.28053	2.02×10^{-17}	9.93×10^{-17}
NRG1	1.117421	0.460111	-1.28012	5.44×10^{-12}	1.46×10^{-11}
MBL2	53.55327	22.17739	-1.27189	7.42×10^{-14}	2.35×10^{-13}
ALB	34010.38	14088.94	-1.27141	6.53×10^{-21}	5.19×10^{-20}
ASPA	1.222286	0.511516	-1.25673	1.83×10^{-17}	9.14×10^{-17}
CD4	24.27378	10.19244	-1.2519	1.84×10^{-18}	1.02×10^{-17}
NR4A2	4.9944	2.106187	-1.24568	1.64×10^{-10}	3.87×10^{-10}
ADH1A	388.2097	164.8505	-1.23568	5.65×10^{-17}	2.56×10^{-16}
HPX	1314.135	561.2385	-1.22743	1.21×10^{-21}	1.06×10^{-20}
BCHE	32.09324	13.77029	-1.22071	5.74×10^{-16}	2.34×10^{-15}
ASS1	584.6253	252.8418	-1.20928	1.11×10^{-18}	6.31×10^{-18}
ATF3	20.57439	9.029725	-1.1881	1.07×10^{-11}	2.79×10^{-11}
ACADS	72.10092	32.05869	-1.1693	9.35×10^{-22}	8.24×10^{-21}
CYP3A4	765.7785	344.3797	-1.15293	1.69×10^{-15}	6.47×10^{-15}
PLG	396.39	178.506	-1.15095	1.74×10^{-19}	1.09×10^{-18}
ALDH2	154.9258	71.34768	-1.11864	1.47×10^{-22}	1.56×10^{-21}
FCGR2B	1.750342	0.811006	-1.10985	1.30×10^{-18}	7.32×10^{-18}
ETFDH	23.2411	10.91094	-1.0909	8.20×10^{-22}	7.40×10^{-21}
FOXO1	9.479037	4.497195	-1.07571	4.28×10^{-15}	1.58×10^{-14}
IL1B	0.87439	0.418351	-1.06356	2.07×10^{-10}	4.81×10^{-10}
DUSP1	210.2614	101.3657	-1.05261	6.88×10^{-14}	2.19×10^{-13}
CYP4F2	60.32353	29.46837	-1.03355	6.21×10^{-15}	2.22×10^{-14}
ADH1C	476.1443	233.4356	-1.02837	1.14×10^{-12}	3.23×10^{-12}
THBS1	24.21534	11.9694	-1.01657	1.49×10^{-9}	3.20×10^{-9}
BACH2	0.544551	0.269844	-1.01294	4.58×10^{-9}	9.36×10^{-9}
OXT	4.631207	2.29837	-1.01078	4.71×10^{-14}	1.52×10^{-13}
NEDD8	7.672523	15.35176	1.000631	5.10×10^{-25}	1.03×10^{-23}

XRCC6	32.25082	64.58255	1.001809	9.42×10^{-25}	1.74×10^{-23}
CYCS	12.62306	25.3376	1.005218	1.71×10^{-15}	6.51×10^{-15}
MCU	1.745668	3.504194	1.005304	8.77×10^{-16}	3.46×10^{-15}
HK1	1.75653	3.532845	1.008103	0.004998	0.006301
MAPK7	0.853585	1.719582	1.01045	2.03×10^{-15}	7.59×10^{-15}
C12orf65	1.090158	2.198122	1.011735	3.43×10^{-24}	5.69×10^{-23}
CASP3	4.186012	8.440812	1.011805	1.98×10^{-17}	9.76×10^{-17}
GSTM2	0.271785	0.548346	1.01262	0.000818	0.001108
FTL	6020.631	12148.79	1.012826	7.28×10^{-7}	1.24×10^{-6}
OSER1	8.295166	16.74718	1.013575	4.75×10^{-24}	7.57×10^{-23}
BCR	2.067768	4.174982	1.013695	3.40×10^{-19}	2.09×10^{-18}
GLRX2	4.420478	8.938884	1.015892	9.59×10^{-18}	5.00×10^{-17}
HTT	1.888609	3.819331	1.015996	4.74×10^{-20}	3.21×10^{-19}
CALR	254.0986	514.2158	1.016986	1.05×10^{-19}	6.83×10^{-19}
NRF1	1.260008	2.555016	1.019899	3.77×10^{-22}	3.69×10^{-21}
GSN	7.907388	16.05391	1.021652	8.66×10^{-10}	1.89×10^{-9}
SLC2A4	0.759184	1.541625	1.021931	0.001768	0.002305
ANXA11	7.942591	16.13269	1.022305	5.74×10^{-19}	3.42×10^{-18}
TYMP	15.65977	31.83915	1.023739	1.52×10^{-6}	2.54×10^{-6}
ACTN4	24.83112	50.50723	1.02434	3.85×10^{-18}	2.06×10^{-17}
NOSIP	5.228417	10.63971	1.025013	4.07×10^{-19}	2.48×10^{-18}
SDHAF1	6.476827	13.18186	1.025195	3.72×10^{-17}	1.75×10^{-16}
PGK1	25.61274	52.13947	1.025514	2.21×10^{-14}	7.34×10^{-14}
HDAC1	9.672532	19.69688	1.026001	6.77×10^{-19}	3.96×10^{-18}
FLT1	1.413798	2.881854	1.027421	2.78×10^{-10}	6.34×10^{-10}
TYK2	4.675963	9.53731	1.028319	2.85×10^{-23}	3.71×10^{-22}
SUMO2	13.86474	28.29764	1.029261	8.60×10^{-23}	9.66×10^{-22}
APEX1	28.08539	57.56035	1.035256	1.71×10^{-24}	2.97×10^{-23}
CNTF	0.074886	0.153485	1.035334	1.00×10^{-6}	1.70×10^{-6}
MSRB3	0.721985	1.481007	1.036538	0.005393	0.006784
ATR	0.630074	1.292794	1.0369	1.97×10^{-16}	8.51×10^{-16}
VIM	24.91128	51.21489	1.039764	1.08×10^{-8}	2.14×10^{-8}
CASP8	1.376885	2.831424	1.040119	1.52×10^{-15}	5.82×10^{-15}
ATP2A2	7.982901	16.42979	1.041329	2.33×10^{-16}	9.95×10^{-16}
PPARD	3.899033	8.030164	1.042313	1.51×10^{-11}	3.89×10^{-11}
FTH1	139.0993	286.4864	1.042351	8.93×10^{-17}	3.98×10^{-16}
CDK6	1.396347	2.876369	1.042591	0.00698	0.008742
NRAS	5.769457	11.90251	1.044759	7.64×10^{-17}	3.42×10^{-16}
EIF2B4	3.154711	6.514626	1.046174	1.32×10^{-25}	3.33×10^{-24}
CAV1	4.222347	8.723696	1.046894	3.25×10^{-9}	6.76×10^{-9}
SMAD2	0.589431	1.22401	1.05422	5.27×10^{-22}	4.95×10^{-21}
NCF2	1.947159	4.045334	1.054888	0.011789	0.014419
HSP90AA1	68.1895	141.9497	1.057758	6.55×10^{-19}	3.87×10^{-18}
PML	1.502347	3.136546	1.061959	9.29×10^{-18}	4.86×10^{-17}
CDK2	2.471469	5.171433	1.065195	8.63×10^{-12}	2.27×10^{-11}
ANXA5	28.45994	59.5752	1.065779	6.91×10^{-9}	1.39×10^{-8}
CIITA	0.380109	0.798406	1.070709	1.61×10^{-5}	2.46×10^{-5}
HSPA5	110.1952	231.5747	1.071416	1.04×10^{-19}	6.79×10^{-19}
PSIP1	2.446736	5.143867	1.071995	2.83×10^{-10}	6.43×10^{-10}
PDLIM4	0.179219	0.377066	1.073095	0.029405	0.034633
CYP21A2	1.286461	2.71175	1.075816	7.70×10^{-5}	0.000112

LANCL1	3.901339	8.325036	1.093487	1.02×10^{-17}	5.30×10^{-17}
SMAD3	2.665888	5.688797	1.093508	8.62×10^{-13}	2.48×10^{-12}
NDUFS8	9.703497	20.70708	1.093547	2.30×10^{-20}	1.66×10^{-19}
PNKP	3.084106	6.581772	1.093624	3.55×10^{-22}	3.50×10^{-21}
CANX	60.54517	129.9874	1.102288	2.00×10^{-21}	1.71×10^{-20}
PDK1	0.517167	1.11061	1.10265	3.65×10^{-8}	6.89×10^{-8}
RPS27A	57.78428	124.1853	1.103745	1.67×10^{-18}	9.32×10^{-18}
ABL1	3.126812	6.745821	1.109302	1.31×10^{-15}	5.05×10^{-15}
EDNRA	0.458486	0.990257	1.110924	0.000584	0.000797
PLAT	0.921995	1.995591	1.113985	0.002229	0.002884
PSEN2	3.208883	6.948669	1.114665	2.86×10^{-15}	1.06×10^{-14}
SLC17A5	5.288773	11.45679	1.115198	1.05×10^{-13}	3.29×10^{-13}
TMEM161A	7.454212	16.1564	1.115978	4.44×10^{-23}	5.36×10^{-22}
MIR93	0.159233	0.346375	1.121195	0.001425	0.00187
TSPO	16.69909	36.38696	1.123652	7.10×10^{-6}	1.11×10^{-5}
CD46	11.0898	24.19239	1.12532	1.83×10^{-17}	9.14×10^{-17}
ITGAM	0.815013	1.778723	1.125947	0.018833	0.022529
BRF2	0.757303	1.653822	1.126861	2.05×10^{-14}	6.86×10^{-14}
TPM1	4.562436	10.00161	1.132356	7.63×10^{-14}	2.41×10^{-13}
DNM1L	2.253031	4.941662	1.133129	3.29×10^{-20}	2.30×10^{-19}
KCNJ2	0.198486	0.435469	1.133532	2.25×10^{-7}	4.01×10^{-07}
TALDO1	32.76224	72.10119	1.137989	2.81×10^{-18}	1.53×10^{-17}
FCGR2A	1.090754	2.404624	1.140486	1.32×10^{-5}	2.03×10^{-5}
ADSL	3.823004	8.444662	1.143333	6.03×10^{-24}	9.09×10^{-23}
STK25	4.234304	9.369389	1.14583	3.92×10^{-26}	1.28×10^{-24}
MTHFR	0.900435	2.003568	1.153877	1.64×10^{-16}	7.15×10^{-16}
PON2	20.78854	46.39852	1.15829	2.85×10^{-16}	1.19×10^{-15}
LBR	7.318591	16.34688	1.159377	2.60×10^{-16}	1.10×10^{-15}
ENO1	103.3429	231.5602	1.163949	1.92×10^{-12}	5.35×10^{-12}
TSC2	2.495921	5.605779	1.167343	4.51×10^{-22}	4.34×10^{-21}
HSPA14	1.368904	3.091901	1.175473	2.59×10^{-22}	2.65×10^{-21}
CXCR3	0.473737	1.080468	1.189497	2.78×10^{-5}	4.18×10^{-5}
COX6B1	113.7581	259.5607	1.190102	6.13×10^{-19}	3.64×10^{-18}
MT-TL1	2.022034	4.620711	1.192308	0.016623	0.02001
PRKD2	3.041684	6.953447	1.192858	9.22×10^{-24}	1.32×10^{-22}
CSF2	0.016745	0.038458	1.199538	0.015016	0.018172
CPQ	11.39795	26.27987	1.205184	5.05×10^{-21}	4.07×10^{-20}
NEAT1	5.481795	12.6457	1.205927	9.87×10^{-9}	1.95×10^{-8}
MAP2K6	0.479664	1.10668	1.206142	0.00015	0.000212
PPARG	2.18644	5.051845	1.208227	6.56×10^{-11}	1.59×10^{-10}
GRN	43.19446	99.89037	1.209499	9.80×10^{-21}	7.39×10^{-20}
IL1A	0.010728	0.024858	1.212383	0.007353	0.009189
DYNC1H1	4.925103	11.42337	1.213763	1.99×10^{-23}	2.66×10^{-22}
PLA2G7	1.733444	4.023915	1.214959	0.000584	0.000797
MAPKAPK2	12.26646	28.5069	1.216592	8.53×10^{-21}	6.47×10^{-20}
MUTYH	1.111061	2.582636	1.216906	3.59×10^{-21}	3.03×10^{-20}
CAPN3	0.099957	0.232545	1.218126	3.22×10^{-5}	4.81×10^{-5}
CAMK2G	1.382707	3.219621	1.219395	1.02×10^{-22}	1.12×10^{-21}
NR2C2	0.656027	1.528133	1.219943	2.68×10^{-12}	7.42×10^{-12}
HYOU1	12.4821	29.08997	1.220661	4.77×10^{-15}	1.73×10^{-14}
SELENON	5.960445	13.94858	1.226626	3.60×10^{-17}	1.71×10^{-16}

NDUFA1	72.08623	168.9613	1.228897	2.75×10^{-23}	3.62×10^{-22}
HSPA4	10.40937	24.42102	1.230241	4.84×10^{-26}	1.54×10^{-24}
TIA1	2.332126	5.507217	1.239678	4.58×10^{-17}	2.11×10^{-16}
IRF5	1.171044	2.776181	1.245307	4.35×10^{-17}	2.02×10^{-16}
GAPDH	278.5148	661.6961	1.248415	5.02×10^{-20}	3.36×10^{-19}
AGER	0.526634	1.257731	1.25595	1.99×10^{-15}	7.46×10^{-15}
RYR3	0.017021	0.04069	1.257344	6.04×10^{-11}	1.47×10^{-10}
NDUFS6	27.06693	65.15192	1.267276	3.42×10^{-22}	3.41×10^{-21}
PECAM1	4.647443	11.19552	1.268413	1.39×10^{-17}	7.09×10^{-17}
HNRNPA1	25.56838	61.66658	1.270128	1.94×10^{-23}	2.65×10^{-22}
LGALS3	10.92912	26.40711	1.272749	4.98×10^{-6}	7.94×10^{-6}
ALDH3B1	1.041834	2.51889	1.273663	1.40×10^{-6}	2.36×10^{-6}
SIRT6	2.380424	5.755776	1.273792	1.63×10^{-23}	2.31×10^{-22}
CYC1	42.94605	103.9532	1.275336	8.60×10^{-23}	9.66×10^{-22}
LTA	0.109635	0.26539	1.275401	1.78×10^{-5}	2.72×10^{-5}
DAXX	6.123839	14.89647	1.282463	1.47×10^{-24}	2.59×10^{-23}
DYNLL1	18.0639	44.0089	1.284686	7.78×10^{-25}	1.46×10^{-23}
TLR5	0.382716	0.933134	1.28581	6.73×10^{-5}	9.82×10^{-5}
GTPBP3	1.389765	3.396237	1.289097	1.34×10^{-25}	3.33×10^{-24}
CTTN	8.362835	20.48029	1.292172	2.55×10^{-27}	1.33×10^{-25}
HRH1	0.142274	0.349236	1.295531	2.92×10^{-7}	5.13×10^{-7}
SNTA1	5.613178	13.7923	1.296973	5.69×10^{-16}	2.33×10^{-15}
NDUFAF2	5.39847	13.27988	1.29862	5.89×10^{-23}	7.03×10^{-22}
ADORA2A	0.190416	0.468726	1.299588	1.40×10^{-11}	3.61×10^{-11}
PPIA	28.55545	70.35979	1.300985	9.81×10^{-28}	7.03×10^{-26}
MIF	18.53651	45.69843	1.301775	2.78×10^{-13}	8.44×10^{-13}
RPTOR	1.577082	3.888593	1.301991	4.43×10^{-25}	9.07×10^{-24}
TGFB1	5.167008	12.75444	1.303598	0.00252	0.003241
NDUFB9	37.22186	92.18654	1.308406	5.07×10^{-20}	3.38×10^{-19}
PKD1	0.781408	1.93702	1.30969	7.73×10^{-19}	4.50×10^{-18}
GPX2	106.9847	267.7037	1.323232	0.010098	0.01247
HSPA1B	12.06733	30.36593	1.331347	4.69×10^{-8}	8.79×10^{-8}
UCP3	0.063033	0.15887	1.333677	5.34×10^{-14}	1.71×10^{-13}
ALOX12	0.109011	0.275266	1.336353	1.04×10^{-12}	2.96×10^{-12}
HSPG2	2.030108	5.126752	1.336489	1.35×10^{-5}	2.07×10^{-5}
LGALS1	43.45304	110.7029	1.349165	3.02×10^{-9}	6.34×10^{-9}
PARP1	7.888009	20.10034	1.349487	6.82×10^{-23}	8.05×10^{-22}
PTK2	2.18261	5.58232	1.354811	1.97×10^{-23}	2.65×10^{-22}
TXN	102.3445	262.081	1.35658	3.68×10^{-17}	1.74×10^{-16}
CS	5.378785	13.81835	1.361233	1.45×10^{-22}	1.55×10^{-21}
PDE5A	0.19598	0.503872	1.362347	1.58×10^{-6}	2.64×10^{-6}
FUS	5.965309	15.3978	1.368055	3.83×10^{-23}	4.72×10^{-22}
MAPK3	3.657011	9.508188	1.378505	5.73×10^{-26}	1.64×10^{-24}
PFKM	0.866943	2.258566	1.381398	3.07×10^{-6}	4.98×10^{-6}
CDK4	5.450969	14.20822	1.382142	8.35×10^{-19}	4.83×10^{-18}
DDAH2	10.22861	26.83559	1.391538	6.97×10^{-15}	2.47×10^{-14}
NPM1	32.823	86.18958	1.392806	3.94×10^{-24}	6.45×10^{-23}
TRPM2	0.266569	0.700193	1.393243	5.54×10^{-7}	9.60×10^{-7}
OGG1	1.016788	2.676699	1.396436	7.28×10^{-24}	1.07×10^{-22}
NLRP1	0.40596	1.071783	1.400604	1.31×10^{-9}	2.83×10^{-9}
MTR	1.273808	3.363203	1.400688	2.77×10^{-16}	1.16×10^{-15}

HSPA1A	22.60168	60.07114	1.410242	5.02×10^{-12}	1.35×10^{-11}
PRKCD	2.370244	6.311841	1.413025	8.94×10^{-16}	3.52×10^{-15}
CAPN2	4.520563	12.0896	1.419192	3.60×10^{-17}	1.71×10^{-16}
ACHE	0.686225	1.858859	1.437664	0.032861	0.038466
LYRM4	0.896192	2.441398	1.445827	6.92×10^{-24}	1.03×10^{-22}
IDO1	0.444307	1.213901	1.450023	0.000361	0.000498
XRCC1	2.861416	7.848694	1.455723	2.35×10^{-27}	1.29×10^{-25}
HSF1	6.466392	17.74508	1.456387	3.17×10^{-25}	6.99×10^{-24}
SLC22A5	0.373483	1.026769	1.458997	1.70×10^{-23}	2.34×10^{-22}
LMNA	21.41802	59.00949	1.462122	2.92×10^{-23}	3.72×10^{-22}
DGKQ	1.45744	4.030869	1.467654	4.12×10^{-23}	5.03×10^{-22}
PRKD1	0.271513	0.751223	1.468218	0.001408	0.001851
AARS2	1.790609	4.973161	1.473713	1.71×10^{-26}	6.33×10^{-25}
PRKCA	1.366614	3.800266	1.475494	2.33×10^{-19}	1.44×10^{-18}
DMPK	0.874301	2.442741	1.482299	4.40×10^{-17}	2.03×10^{-16}
CHKB	0.422492	1.182077	1.484327	9.12×10^{-19}	5.25×10^{-18}
ACACA	1.393724	3.919558	1.491746	6.37×10^{-20}	4.20×10^{-19}
BBC3	1.24966	3.516938	1.492785	1.16×10^{-19}	7.43×10^{-19}
GJA1	2.160887	6.08789	1.494318	1.75×10^{-10}	4.10×10^{-10}
ADA	0.943393	2.672451	1.502233	1.19×10^{-17}	6.10×10^{-17}
BRCA1	0.351409	0.995991	1.502983	5.86×10^{-12}	1.57×10^{-11}
OSGIN1	22.74322	64.47217	1.50324	1.32×10^{-7}	2.40×10^{-7}
PYCR2	5.378948	15.26389	1.504727	5.48×10^{-28}	4.48×10^{-26}
NOX1	0.146418	0.415607	1.505125	9.70×10^{-15}	3.37×10^{-14}
DDIT3	9.15751	26.01926	1.506553	3.18×10^{-17}	1.54×10^{-16}
IKBKG	1.043792	2.969835	1.508548	9.70×10^{-15}	3.37×10^{-14}
NOL3	2.546747	7.252008	1.509725	1.76×10^{-13}	5.42×10^{-13}
MALAT1	2.157594	6.152177	1.511673	1.04×10^{-15}	4.08×10^{-15}
BAX	7.321618	21.10099	1.527077	7.52×10^{-23}	8.62×10^{-22}
CASP2	0.781201	2.257459	1.530935	8.14×10^{-21}	6.22×10^{-20}
GPX7	1.502034	4.344911	1.532409	0.000302	0.00042
GSTO2	0.303991	0.88077	1.534738	0.001202	0.001589
CACNA1A	0.013225	0.038351	1.535934	2.90×10^{-8}	5.51×10^{-8}
SHC1	12.26508	35.709	1.541731	5.37×10^{-26}	1.61×10^{-24}
CDK5	2.131995	6.231829	1.547452	1.87×10^{-27}	1.08×10^{-25}
ANGPT1	0.156857	0.458629	1.547875	3.95×10^{-8}	7.41×10^{-08}
VDR	0.303731	0.895339	1.55964	1.87×10^{-5}	2.83×10^{-5}
TSC1	0.723672	2.14458	1.567288	2.41×10^{-24}	4.06×10^{-23}
PDGFB	1.440219	4.273008	1.568964	1.84×10^{-18}	1.02×10^{-17}
ATP13A2	1.26297	3.755852	1.57232	2.33×10^{-19}	1.44×10^{-18}
TFRC	3.679605	10.99098	1.578697	1.93×10^{-16}	8.36×10^{-16}
UBQLN4	3.940139	11.77271	1.579128	2.39×10^{-25}	5.59×10^{-24}
HRAS	4.521073	13.56737	1.585403	7.59×10^{-25}	1.45×10^{-23}
NPPA	0.03849	0.116257	1.594779	6.21×10^{-7}	1.07×10^{-6}
STIP1	8.394324	25.5146	1.603837	1.37×10^{-26}	5.40×10^{-25}
CLIC1	32.75503	99.90018	1.608771	3.93×10^{-18}	2.10×10^{-17}
BAK1	2.432961	7.457171	1.615915	1.55×10^{-17}	7.78×10^{-17}
ABCD1	2.721367	8.418066	1.629157	5.05×10^{-21}	4.07×10^{-20}
ACE	0.280269	0.869131	1.63276	1.28×10^{-15}	4.96×10^{-15}
HSP90AB1	114.1782	354.4065	1.634118	3.53×10^{-26}	1.19×10^{-24}
SPARC	29.50254	91.8974	1.639185	2.35×10^{-16}	1.00×10^{-15}

BSG	38.8006	120.901	1.639675	5.16×10^{-26}	1.60×10^{-24}
SMARCA4	2.346068	7.314386	1.640492	4.92×10^{-27}	2.17×10^{-25}
AGRN	5.444517	17.04347	1.646343	1.97×10^{-15}	7.41×10^{-15}
FASN	26.76458	84.17382	1.653046	1.74×10^{-12}	4.88×10^{-12}
PLCG1	1.26078	3.995511	1.664063	1.77×10^{-21}	1.54×10^{-20}
SLC7A1	0.447873	1.433182	1.678059	2.47×10^{-05}	3.73×10^{-5}
TAZ	1.723546	5.520824	1.679503	7.52×10^{-29}	2.84×10^{-26}
TLR9	0.003203	0.010297	1.68454	2.65×10^{-6}	4.35×10^{-6}
MATR3	0.033426	0.108406	1.697405	5.40×10^{-22}	4.99×10^{-21}
TUBA1B	8.538006	27.87822	1.707167	1.04×10^{-17}	5.39×10^{-17}
CTLA4	0.179603	0.589593	1.714908	6.29×10^{-6}	9.90×10^{-6}
CPT1B	0.121235	0.39859	1.717098	1.74×10^{-14}	5.85×10^{-14}
GSTA4	3.882656	12.89632	1.731844	2.91×10^{-16}	1.21×10^{-15}
ANGPT2	0.342389	1.139631	1.734857	2.11×10^{-14}	7.03×10^{-14}
SQSTM1	29.98683	99.91757	1.736409	9.02×10^{-17}	4.01×10^{-16}
ABCC1	0.707154	2.380374	1.751092	3.82×10^{-6}	6.16×10^{-6}
MIR210	0.098696	0.332422	1.751958	0.001846	0.002404
PCNA	14.13424	47.90249	1.760906	3.47×10^{-25}	7.37×10^{-24}
DNASE1	0.148796	0.505421	1.764154	1.19×10^{-22}	1.29×10^{-21}
KCNQ1	0.759955	2.5843	1.765787	0.011344	0.013934
SCN4B	0.139019	0.476334	1.776694	1.66×10^{-14}	5.62×10^{-14}
CHKA	3.366625	11.5937	1.783966	5.27×10^{-22}	4.95×10^{-21}
NME1	5.125199	17.66288	1.785041	4.09×10^{-24}	6.60×10^{-23}
GPX8	0.394961	1.365423	1.789566	1.64×10^{-09}	3.50×10^{-9}
GLS	1.20176	4.164693	1.793062	3.87×10^{-12}	1.05×10^{-11}
SLC1A3	0.376073	1.307823	1.798083	1.13×10^{-11}	2.95×10^{-11}
FRZB	0.835804	2.910029	1.799797	2.47×10^{-10}	5.65×10^{-10}
KCNT1	0.003929	0.013749	1.807154	2.66×10^{-7}	4.71×10^{-7}
PPOX	1.196744	4.192724	1.808773	1.44×10^{-29}	1.65×10^{-26}
GLA	3.241931	11.36255	1.809361	1.20×10^{-24}	2.15×10^{-23}
PDGFRB	2.879883	10.19559	1.823863	7.33×10^{-17}	3.31×10^{-16}
TRAF2	2.172886	7.704197	1.826032	3.25×10^{-27}	1.62×10^{-25}
ANXA2	11.02102	39.29083	1.833935	6.77×10^{-19}	3.96×10^{-18}
SERPINH1	6.279245	22.40272	1.835011	1.75×10^{-20}	1.27×10^{-19}
CACNA1C	0.048833	0.176691	1.855296	8.59×10^{-11}	2.05×10^{-10}
SRC	1.47643	5.368635	1.862442	2.16×10^{-10}	4.98×10^{-10}
PIK3R2	0.082801	0.305292	1.882465	2.52×10^{-19}	1.55×10^{-18}
SLC2A1	0.680047	2.534008	1.897715	3.92×10^{-5}	5.80×10^{-5}
NDRG1	6.834797	25.56367	1.903125	7.08×10^{-12}	1.88×10^{-11}
UCN	0.428253	1.604284	1.905396	8.59×10^{-16}	3.43×10^{-15}
DNMT1	1.168587	4.380206	1.906233	7.50×10^{-21}	5.85×10^{-20}
MICB	0.463991	1.750292	1.915426	1.08×10^{-15}	4.23×10^{-15}
HSPA6	1.127275	4.298705	1.931063	0.000793	0.001076
IRAK1	7.955855	30.66443	1.946477	6.88×10^{-26}	1.88×10^{-24}
MECOM	0.207892	0.801388	1.946663	8.22×10^{-14}	2.59×10^{-13}
BDKRB2	0.245237	0.959321	1.967835	8.58×10^{-08}	1.59×10^{-7}
CDKN2B	0.543072	2.134847	1.974919	4.38×10^{-18}	2.31×10^{-17}
BDNF	0.014482	0.057163	1.980833	0.000379	0.000521
PLA2G6	0.580481	2.296062	1.98384	5.46×10^{-24}	8.34×10^{-23}
NES	1.85052	7.350038	1.989821	6.16×10^{-22}	5.64×10^{-21}
MAPK11	0.83053	3.321739	1.999835	1.08×10^{-19}	6.98×10^{-19}

TGFB2	0.242074	0.973947	2.008395	9.02×10^{-5}	0.00013
MSH2	0.977397	3.939985	2.011174	8.59×10^{-26}	2.29×10^{-24}
ARG2	0.495924	2.010794	2.019573	7.62×10^{-5}	0.000111
PLAU	0.978175	3.987996	2.0275	4.04×10^{-15}	1.49×10^{-14}
GBA	5.56523	22.70088	2.028235	3.64×10^{-28}	4.17×10^{-26}
CASQ2	0.110269	0.449845	2.028407	7.03×10^{-11}	1.70×10^{-10}
SRXN1	0.361867	1.488914	2.040729	5.06×10^{-15}	1.82×10^{-14}
SULT1A3	0.004162	0.017222	2.048717	1.82×10^{-9}	3.88×10^{-9}
NOS2	0.097262	0.402505	2.049054	5.21×10^{-13}	1.55×10^{-12}
VWF	1.87895	7.784544	2.050686	2.34×10^{-17}	1.14×10^{-16}
TXNRD1	6.763141	28.10662	2.055145	1.93×10^{-17}	9.60×10^{-17}
BRCA2	0.069395	0.288753	2.056943	4.81×10^{-19}	2.90×10^{-18}
ACTA1	0.053393	0.224811	2.073989	7.24×10^{-11}	1.74×10^{-10}
MB	0.168985	0.719464	2.090026	0.011584	0.014199
MMP14	5.296398	23.13895	2.12724	7.85×10^{-16}	3.15×10^{-15}
MIR126	0.24558	1.07377	2.128419	3.56×10^{-11}	8.82×10^{-11}
MAP2	0.462856	2.031434	2.133864	6.30×10^{-10}	1.40×10^{-9}
ALOX15	0.021905	0.096189	2.134597	8.16×10^{-6}	1.27×10^{-5}
PDCD1	0.292666	1.299295	2.1504	0.01699	0.020431
CYP1B1	1.337916	5.949697	2.152829	0.006929	0.008687
TREM2	1.200667	5.368336	2.160639	4.91×10^{-13}	1.47×10^{-12}
PRKCG	0.003641	0.016335	2.165414	0.009676	0.011988
CALB1	0.01974	0.090562	2.197775	0.001535	0.00201
OXTR	0.145185	0.680815	2.22937	1.42×10^{-7}	2.57×10^{-7}
MIR25	0.570133	2.686576	2.236397	3.69×10^{-17}	1.74×10^{-16}
HSPB1	71.35463	339.0672	2.248492	7.65×10^{-24}	1.11×10^{-22}
SNCAIP	0.115128	0.556	2.271849	5.41×10^{-6}	8.61×10^{-06}
PLCB1	0.225676	1.091509	2.274001	4.77×10^{-14}	1.54×10^{-13}
SCARA3	1.131286	5.535768	2.29082	8.34×10^{-10}	1.83×10^{-9}
MMP3	0.04299	0.212156	2.303061	0.011913	0.014555
MAPK13	0.578055	2.884652	2.31912	0.00835	0.010401
GRM1	0.002632	0.013361	2.343928	0.002786	0.003579
LOX	0.361599	1.837614	2.345371	9.00×10^{-12}	2.37×10^{-11}
PDGFRL	0.159697	0.823099	2.365731	8.68×10^{-16}	3.45×10^{-15}
BGLAP	0.110832	0.592322	2.418008	4.44×10^{-21}	3.63×10^{-20}
SYP	0.091164	0.489053	2.423462	3.99×10^{-20}	2.72×10^{-19}
TNFSF4	0.190816	1.024079	2.42407	1.01×10^{-18}	5.74×10^{-18}
BMP4	0.775733	4.170191	2.426482	3.34×10^{-8}	6.32×10^{-08}
CHEK1	0.246453	1.330717	2.43282	3.85×10^{-25}	8.02×10^{-24}
NUDT1	0.946982	5.227625	2.464746	1.83×10^{-26}	6.55×10^{-25}
PRKAA2	0.279372	1.628446	2.543237	2.30×10^{-10}	5.29×10^{-10}
REN	0.264428	1.557061	2.557881	0.001046	0.001394
TPH1	0.011207	0.069008	2.622363	6.41×10^{-10}	1.42×10^{-9}
PRPH	0.003532	0.021946	2.63541	0.030897	0.036279
KCNJ5	0.156276	0.974231	2.64017	1.42×10^{-17}	7.21×10^{-17}
PKM	4.018636	25.24353	2.651136	1.86×10^{-14}	6.27×10^{-14}
ACSL4	7.321489	46.12192	2.655243	3.57×10^{-12}	9.80×10^{-12}
GLUL	58.17993	366.9452	2.656971	0.000191	0.000268
SCN4A	0.034643	0.223416	2.689077	4.04×10^{-21}	3.36×10^{-20}
CD34	0.739825	4.800344	2.697881	9.90×10^{-29}	2.84×10^{-26}
POU5F1	0.115606	0.753411	2.704226	3.69×10^{-18}	1.98×10^{-17}

STK39	0.470728	3.076311	2.708237	6.27×10^{-14}	2.00×10^{-13}
MMP9	1.388571	9.153536	2.720729	2.14×10^{-8}	4.14×10^{-8}
ITGA2	0.202899	1.339145	2.722479	2.68×10^{-14}	8.80×10^{-14}
MAPK12	0.174319	1.201013	2.784453	9.53×10^{-19}	5.46×10^{-18}
DRD4	0.155276	1.075832	2.792546	1.06×10^{-14}	3.63×10^{-14}
DNAH8	0.002715	0.020204	2.895862	3.50×10^{-9}	7.26×10^{-9}
CCNF	0.195257	1.489003	2.930903	1.57×10^{-27}	1.00×10^{-25}
IGF2BP2	0.520433	3.98315	2.936124	1.22×10^{-7}	2.22×10^{-7}
FANCD2	0.131555	1.016427	2.949769	2.65×10^{-25}	6.07×10^{-24}
FMO1	0.209437	1.621168	2.952447	0.003981	0.005058
LPL	0.14158	1.102973	2.961704	1.86×10^{-21}	1.60×10^{-20}
RAD51	0.141944	1.2032	3.083487	3.17×10^{-25}	6.99×10^{-24}
EZH2	0.342823	2.947812	3.104109	4.59×10^{-28}	4.38×10^{-26}
DUOX1	0.056932	0.494106	3.117501	5.88×10^{-20}	3.90×10^{-19}
SCN5A	0.003028	0.026658	3.137971	1.43×10^{-5}	2.19×10^{-5}
PYCR1	0.861568	8.121218	3.236659	9.35×10^{-6}	1.45×10^{-5}
ABCC8	0.010043	0.103051	3.359152	0.001226	0.001619
G6PD	1.311715	13.58215	3.372185	6.03×10^{-25}	1.19×10^{-23}
AURKA	0.65543	6.892745	3.394565	4.06×10^{-28}	4.23×10^{-26}
CNR1	0.018329	0.201174	3.456231	0.001196	0.001583
NOX4	0.021637	0.24261	3.487096	1.94×10^{-28}	3.92×10^{-26}
GRIN2A	0.0086	0.099026	3.525448	0.000892	0.001197
MMP1	0.168919	1.998116	3.564235	2.20×10^{-9}	4.67×10^{-9}
CACNA1S	0.002171	0.026022	3.583562	1.06×10^{-11}	2.77×10^{-11}
GAL	0.033022	0.431447	3.707689	0.015201	0.018376
MAPT	0.042747	0.566576	3.728369	3.34×10^{-25}	7.22×10^{-24}
GAP43	0.011057	0.150101	3.76287	1.35×10^{-9}	2.90×10^{-9}
CACNB4	0.005895	0.081635	3.791652	4.89×10^{-15}	1.77×10^{-14}
GRIN1	0.00215	0.029834	3.794455	6.27×10^{-8}	1.17×10^{-7}
TACR1	0.015485	0.216399	3.804787	0.000213	0.000299
CCNB1	0.577324	9.413162	4.027226	2.73×10^{-28}	3.92×10^{-26}
TP73	0.030832	0.529801	4.102942	2.96×10^{-23}	3.73×10^{-22}
MKI67	0.159353	2.813747	4.142196	1.44×10^{-26}	5.51×10^{-25}
CCNA2	0.282182	5.21444	4.207815	3.92×10^{-27}	1.87×10^{-25}
CDK1	0.228708	4.319807	4.239388	7.09×10^{-28}	5.41×10^{-26}
E2F1	0.445127	9.101468	4.35381	1.32×10^{-27}	8.90×10^{-26}
FOXM1	0.191186	3.995444	4.385308	4.85×10^{-27}	2.17×10^{-25}
UNC13A	0.005135	0.108319	4.398831	2.73×10^{-12}	7.55×10^{-12}
SOX2	0.019622	0.417033	4.409603	0.000866	0.001167
CDKN3	0.214098	5.262967	4.619531	9.90×10^{-29}	2.84×10^{-26}
CDKN2A	0.162719	4.09759	4.65432	1.87×10^{-25}	4.56×10^{-24}
IL11	0.018755	0.489814	4.706871	7.00×10^{-9}	1.40×10^{-8}
BIRC5	0.250505	7.049579	4.814625	2.35×10^{-28}	3.92×10^{-26}
MT3	0.017155	0.505604	4.881317	0.000988	0.00132
SLC7A11	0.030108	0.889909	4.885429	1.65×10^{-19}	1.05×10^{-18}
CYP17A1	0.764053	22.79083	4.898637	2.78×10^{-5}	4.18×10^{-5}
TYRP1	0.0086	0.267725	4.960195	0.000375	0.000517
SPP1	10.48235	336.86	5.006114	2.72×10^{-7}	4.80×10^{-7}
CDC25C	0.050896	1.660882	5.028265	2.62×10^{-28}	3.92×10^{-26}
UCN2	0.002493	0.081838	5.036608	1.49×10^{-12}	4.18×10^{-12}
LCN2	3.786378	138.255	5.190369	1.91×10^{-10}	4.46×10^{-10}

ACTN2	0.052553	2.040419	5.278942	3.77×10^{-9}	7.80×10^{-9}
NQO1	1.459665	57.95383	5.311194	6.82×10^{-16}	2.75×10^{-15}
SLC6A3	0.003172	0.138398	5.447314	0.000121	0.000172
GAD1	0.002933	0.132777	5.500359	4.82×10^{-14}	1.55×10^{-13}
UCHL1	0.161748	7.410817	5.517809	8.24×10^{-8}	1.53×10^{-7}
EGF	0.005062	0.244473	5.593952	1.10×10^{-5}	1.69×10^{-5}
ALDH3A1	0.752937	44.07999	5.871452	0.000343	0.000475
HTR3A	0.006152	0.407324	6.049049	0.000548	0.000749
PDIA2	0.015381	1.664479	6.757734	1.94×10^{-14}	6.49×10^{-14}
CYP19A1	0.001926	0.354051	7.522496	2.10×10^{-10}	4.85×10^{-10}
IGF2BP1	0.009142	1.857593	7.666682	2.86×10^{-16}	1.19×10^{-15}
CHGA	0.007962	2.125302	8.060408	7.34×10^{-13}	2.13×10^{-12}
TERT	0.001912	1.369339	9.483907	5.48×10^{-26}	1.61×10^{-24}
COL2A1	0.003647	3.755195	10.00783	1.66×10^{-7}	3.00×10^{-7}

Table S2. 146 prognostic-associated candidate DEOSGs identified by univariate Cox regression analysis in HCC.

id	HR	HR.95L	HR.95H	*p*-value
ANXA5	1.358915	1.154538	1.599471	0.000226
SLC17A5	1.359152	1.079326	1.711526	0.009081
ABL1	1.377115	1.038252	1.826575	0.026388
IRF5	1.495095	1.089937	2.050861	0.012631
PPARG	1.262734	1.052614	1.514799	0.012
PGK1	1.447938	1.178883	1.778401	0.000417
RAD51	1.562273	1.19405	2.04405	0.001141
MKI67	1.459372	1.210287	1.759721	7.53×10^{-5}
MATR3	2.957867	1.434716	6.098054	0.003305
GADD45B	0.842328	0.728094	0.974485	0.021024
DYNLL1	1.909708	1.358364	2.684835	0.000198
PRKCD	1.616056	1.281551	2.037871	4.98×10^{-5}
ADSL	1.991414	1.469914	2.697933	8.73×10^{-6}
CLIC1	1.312803	1.108283	1.555064	0.001634
FANCD2	1.652802	1.236506	2.209254	0.000689
HSPA4	1.837623	1.292551	2.612552	0.0007
NRF1	1.931869	1.200278	3.109378	0.006693
RYR3	3.621311	1.114584	11.76573	0.032322
CS	1.308937	1.008195	1.699391	0.043255
LOX	1.419518	1.18827	1.69577	0.000113
GRN	1.402582	1.111381	1.770082	0.00438
SMAD2	2.436764	1.482331	4.005731	0.000445
EGF	1.572648	1.145657	2.158781	0.00509
GLRX2	1.549088	1.148339	2.089693	0.004163
GTPBP3	1.466311	1.003067	2.143494	0.04818
GPX7	1.193388	1.029175	1.383802	0.019247
PLCG1	1.384955	1.047557	1.831023	0.022248
MIR210	1.929318	1.306427	2.849197	0.000954
CDKN2B	1.598726	1.257305	2.032859	0.000129
ABCD1	1.310686	1.037205	1.656278	0.023457
DYNC1H1	2.06329	1.512099	2.815401	4.93×10^{-6}
NDRG1	1.323633	1.155505	1.516224	5.22×10^{-5}
MSH2	1.768688	1.34212	2.330832	5.13×10^{-5}
FOXM1	1.389547	1.170408	1.649716	0.000172
ENO1	1.647956	1.367908	1.985338	1.47×10^{-7}
PYCR1	1.150033	1.034642	1.278294	0.009563
MUTYH	1.986144	1.458558	2.704568	1.32×10^{-5}

CYP2C9	0.85788	0.798851	0.921272	2.50×10^{-5}
CYP4F2	0.901753	0.820653	0.990869	0.031497
CDK4	1.637513	1.302676	2.058416	2.38×10^{-5}
CDK1	1.431143	1.208416	1.694921	3.28×10^{-5}
NDUFAF2	1.586521	1.213577	2.074073	0.000736
CASP2	1.879512	1.354532	2.607962	0.00016
CDC25C	1.486003	1.192542	1.851678	0.000418
BAK1	1.408167	1.1382	1.742167	0.001622
G6PD	1.430097	1.267852	1.613104	5.79×10^{-9}
IL1RN	0.863516	0.750288	0.99383	0.04073
SRC	1.279088	1.076021	1.520477	0.00526
LCAT	0.757744	0.664584	0.863963	3.40×10^{-5}
CYP2C8	0.918558	0.855642	0.986099	0.018943
PLG	0.908452	0.837538	0.98537	0.020593
STK39	1.322125	1.13226	1.543827	0.000415
EIF2B4	2.469555	1.65345	3.688469	1.00×10^{-5}
APEX1	1.822289	1.278537	2.597295	0.000904
AGRN	1.345317	1.13841	1.589829	0.000499
ALDH2	0.712176	0.599524	0.845996	0.000112
CHGA	1.270068	1.065235	1.514287	0.007717
LYRM4	1.466925	1.073206	2.005084	0.016259
ABCC1	1.404013	1.168157	1.68749	0.000299
SUMO2	1.614444	1.161933	2.243184	0.004313
SQSTM1	1.410614	1.180321	1.685839	0.000155
TXNRD1	1.384358	1.18603	1.615851	3.75×10^{-5}
TUBA1B	1.340255	1.110288	1.617854	0.002294
GBA	1.359702	1.08046	1.711113	0.008799
DNMT1	1.465779	1.155662	1.859115	0.001617
TNFSF4	1.372235	1.070047	1.759762	0.012651
E2F1	1.24359	1.074934	1.438708	0.003371
GAD1	1.646222	1.201276	2.255975	0.001931
BRCA2	2.041706	1.074219	3.880552	0.029371
CDK5	1.538465	1.121869	2.109762	0.007502
DNM1L	1.728881	1.207259	2.475881	0.002809
IRAK1	1.481704	1.192581	1.84092	0.000385
GLS	1.302999	1.078272	1.574561	0.00614
CHEK1	1.844469	1.391938	2.444122	2.02×10^{-5}
BIRC5	1.359539	1.175915	1.571837	3.34×10^{-5}
AURKA	1.295564	1.094949	1.532935	0.002555
NRAS	1.871932	1.398004	2.506522	2.56×10^{-5}
MTHFR	1.585269	1.103222	2.277945	0.012736
CDK2	1.585663	1.220746	2.059663	0.000551
ADH1A	0.894158	0.831372	0.961686	0.002598
LPA	0.793294	0.671201	0.937596	0.006615
IGF2BP2	1.175586	1.028783	1.343337	0.017458
FUS	1.393606	1.012654	1.917868	0.041635
PSIP1	1.35831	1.064438	1.733316	0.013816
HRAS	1.327302	1.072679	1.642366	0.009172
CDKN2A	1.261015	1.088089	1.461424	0.002057
ACACA	1.677837	1.265134	2.225169	0.000327
STIP1	2.049954	1.527446	2.751201	1.74×10^{-6}
MAPKAPK2	1.533819	1.142942	2.058373	0.004369
LBR	1.311883	1.032249	1.66727	0.026455
CCNA2	1.366522	1.169644	1.59654	8.35×10^{-5}
ACADS	0.715422	0.569394	0.8989	0.00404
HSP90AB1	1.393185	1.092512	1.776606	0.007511
HTT	1.49605	1.009714	2.216634	0.044627
FTL	1.199219	1.013911	1.418394	0.033898
PLCB1	1.471038	1.127751	1.91882	0.004418
MAPK7	2.11323	1.452869	3.07374	9.08×10^{-5}

CYP3A4	0.940115	0.891044	0.991888	0.02396
SHC1	1.466223	1.130962	1.900867	0.003864
CCNF	1.920784	1.436097	2.569055	1.09×10^{-5}
HPX	0.871392	0.808445	0.93924	0.00032
SIRT6	1.547355	1.136906	2.105985	0.005506
HSF1	1.403788	1.092216	1.80424	0.008077
PPIA	1.792664	1.314749	2.444303	0.000224
STK25	2.101575	1.414147	3.123167	0.000238
PLA2G7	1.229079	1.062801	1.421371	0.005415
HSP90AA1	1.585189	1.245279	2.017881	0.000183
ASS1	0.830684	0.719038	0.959666	0.011768
TRAF2	1.482326	1.133737	1.938095	0.004007
TALDO1	1.451032	1.167372	1.803619	0.000795
MCU	1.689514	1.219136	2.341377	0.001632
SLC2A1	1.535566	1.32953	1.77353	5.39×10^{-9}
ESR1	0.685555	0.50075	0.938564	0.018494
UCN	1.284063	1.022264	1.612908	0.031615
SMARCA4	1.595091	1.155792	2.20136	0.004499
NPM1	1.621484	1.265119	2.078232	0.000135
CDKN3	1.297258	1.106418	1.521014	0.001348
CASP8	1.558447	1.099408	2.20915	0.012691
GLA	1.426714	1.157085	1.759172	0.000884
OGG1	1.700648	1.220309	2.370057	0.001714
UBQLN4	1.481527	1.133229	1.936875	0.004045
SLC7A1	1.455776	1.18089	1.79465	0.000436
NEDD8	1.586826	1.118888	2.250463	0.009594
HDAC1	2.015848	1.493873	2.720208	4.54×10^{-6}
HSPA14	2.352946	1.609154	3.440538	1.02×10^{-5}
EZH2	1.819988	1.4277	2.320065	1.33×10^{-6}
SRXN1	1.546388	1.286641	1.858572	3.38×10^{-6}
ATR	2.157085	1.315948	3.535864	0.002297
FOXO1	0.712516	0.547226	0.927732	0.011836
NUDT1	1.29742	1.078122	1.561326	0.005848
BRCA1	1.665841	1.217374	2.279517	0.001427
XRCC6	1.677464	1.221557	2.303524	0.00139
CCNB1	1.478463	1.251037	1.747231	4.47×10^{-6}
CANX	1.352257	1.033025	1.770141	0.02806
NME1	1.324497	1.083162	1.619601	0.006175
SLC22A5	1.502934	1.004334	2.249065	0.047592
C12orf65	2.075883	1.324448	3.25365	0.001445
PCNA	1.467673	1.172139	1.83772	0.000824
ADH1C	0.909717	0.85388	0.969206	0.003414
MAPK3	1.541989	1.167651	2.036337	0.00227
MECOM	1.613397	1.186159	2.19452	0.002306
PPARD	1.338377	1.044279	1.715302	0.021324
PTK2	1.433704	1.059143	1.940728	0.019707
BSG	1.354997	1.127263	1.628739	0.001212
ALB	0.910296	0.842541	0.9835	0.01724
GAPDH	1.495792	1.214631	1.842036	0.00015

Table S3. The regression coefficient of five candidate DEOSGs genes.

ID	Co-ef	HR	HR.95L	HR.95H	pvalue
LOX	0.246096	1.279023	1.065033	1.536007	0.008428
CYP2C9	-0.08318	0.920189	0.848452	0.99799	0.044585
EIF2B4	0.551061	1.735093	1.142665	2.634673	0.009717
EZH2	0.356187	1.427874	1.096691	1.859069	0.008158
SRXN1	0.352746	1.42297	1.182244	1.712712	0.000191

Table S4. Primers of genes for RT–Qpcr.

Primer Name	5' sequence to 3'
GAPDH Forward Primer	AATTCCATGGCACCGTCAAG
GAPDH Reverse Primer	ATCGCCCCACTTGATTTTGG
CYP2C9 Forward Primer	GCCTGAAACCCATAGTGGTG
CYP2C9 Reverse Primer	GGGGCTGCTCAAAATCTTGATG
EIF2B4 Forward Primer	CAGAGAACTGCCAGAATCGGG
EIF2B4 Reverse Primer	GTTCGGCCTTACTCCGACC
EZH2 Forward Primer	AATCAGAGTACATGCGACTGAGA
EZH2 Reverse Primer	GCTGTATCCTTCGCTGTTTCC
SRXN1 Forward Primer	CAGGGAGGTGACTACTTCTACTC
SRXN1 Reverse Primer	CAGGTACACCCTTAGGTCTGA
LOX Forward Primer	CTATGACCTGCTTGATGCCAAC
LOX Reverse Primer	TAACAGCCAGGACTCAATCCC
